# NADPH Oxidase-Mediated Testicular Oxidative Imbalance Regulates the TXNIP/NLRP3 Inflammasome Axis Activation after Ischemia Reperfusion Injury

**DOI:** 10.3390/antiox12010145

**Published:** 2023-01-07

**Authors:** Duaah Almarzouq, May Al-Maghrebi

**Affiliations:** Department of Biochemistry, College of Medicine, Kuwait University, Safat 13110, Kuwait

**Keywords:** NADPH oxidase, oxidative stress, NLRP3 inflammasome, testis, ischemia reperfusion injury

## Abstract

Oxidative stress, inflammation and germ cell death are the main characteristics of testicular ischemia reperfusion injury (tIRI), which is considered as the underlying mechanism for testicular torsion and detorsion. The study aimed to examine the effect of tIRI-activated NADPH oxidase (NOX) on the expression of the NLRP3 inflammasome pathway components. Three groups of male Sprague–Dawley rats (*n* = 12 each) were studied: sham, unilateral tIRI only and tIRI treated with apocynin, a NOX-specific inhibitor. The tIRI rat model was subjected to 1 h of ischemia followed by 4 h of reperfusion. H&E staining, real time PCR, biochemical assays, and Western blot were utilized to evaluate spermatogenic damage, gene expression, oxidative stress markers, and NLRP3 pathway components, respectively. As a result of tIRI, decreased total antioxidant capacity and suppressed activities of superoxide dismutase and catalase were associated with spermatogenic arrest. The components of the NLRP3 inflammasome pathway (TXNIP, NLRP3, ASC, caspase-1, GSDMD, MMP-9) were upregulated transcriptionally and post-transcriptionally during tIRI. In parallel, tissue inflammation was demonstrated by a marked increase in the concentrations of myeloperoxidase, IL-1β, and IL-18. Apocynin treatment prevented testicular oxidative stress and inflammation. Thus, NOX inhibition by apocynin prevented ROS accumulation, proinflammatory cytokine overexpression and NLRP3 inflammasome activation during tIRI.

## 1. Introduction

Maintenance of a balanced intracellular antioxidant/oxidant system is crucial for the health of the male reproductive system since most male fertility issues are associated with oxidative stress (OS) [1]. One main function of the testis is spermatogenesis, a complex and dynamic process that is also energetically expensive. A physiological side product of energy generation for spermatogenesis is oxygen reactive species (ROS), which are faithfully neutralized by the intracellular antioxidant system [2]. However, some medical pathologies were identified as causatives of non-physiological generation and accumulation of intracellular ROS leading to testicular dysfunction and arrested spermatogenesis [3]. One of these conditions is testicular torsion (TT) and its current surgical treatment option, detorsion (D) [4]. In TTD, both torsion (ischemia) and detorsion (reperfusion) result in the exaggerated generation of ROS due to increased electron leakage from the mitochondria and amplified ROS generation upon reoxygenation, respectively [4]. ROS are mainly produced via the mitochondrial oxidative phosphorylation (OXPHOS) system through enzymatic reactions catalyzed by cyclooxygenases, NADPH oxidases (NOXs), xanthine oxidases, and lipoxygenases [2]. It is highly possible that these enzymes may work collaboratively during reperfusion to create a massive oxidative insult in the injured tissue.

NOX5 was identified in both testes and spermatozoa as the major source of ROS, more specifically superoxide (O_2_^●−^) radicals [5]. The O_2_^●−^ radical is an initial and central ROS that is generated by NOX upon catalyzing one electron reduction of molecular oxygen (O_2_) to generate O_2_^●−^. In humans, the oxidative role of NOX was reported in male fertility conditions like asthenozoospermia and teratozoospermia [6,7]. It was also observed that increased NOX activity resulted in significant testicular damage, which was restored by antioxidant treatment [8]. The role of NOX in ROS production was studied in a rat model for unilateral testicular ischemia reperfusion injury (tIRI), where NOX activity was inhibited using its specific inhibitor, apocynin [9]. Such treatment resulted in normalization of tIRI-induced lipid and protein peroxidation, spermatogenic and DNA damages, and germ cell apoptosis (GCA). The levels of the main intracellular antioxidant enzyme superoxide dismutase (SOD) were also reduced during tIRI implicating the imbalance between the antioxidant/oxidant systems during tIRI in favor of OS.

Inflammation is another major phenotype of tIRI that is suggested to be downstream of OS. NOX-generated ROS has been identified as a key factor in the activation of the cytosolic NOD-like receptors-3 (NLRP3) inflammasome suggesting its regulation by NOX activity [10]. NLRP3 is a multi-protein complex and one of the most significant and investigated inflammasomes [11]. Its activation has been associated with reduced sperm count and sperm defects identified in several male infertility situations like genital tract infections and varicocele [12]. It was suggested that ROS accumulation triggers the sensor NLRP3 protein to interact with the adaptor apoptosis-associated spec-like protein (ASC) and the effector caspase-1 forming the active NLRP3 inflammasome complex [13]. The inflammasome complex activates the downstream cytokine interleukin-18 (IL-18) and Matrix metalloproteinase-9 (MMP9), which releases IL-1β. The latter is also known to be cleaved by caspase 8, which has a non-apoptotic function in inflammasome priming [14]. On the other hand, caspase-1 cleaves the effector gasdermin D (GSDMD), which forms a transmembrane pore allowing the release of IL-1β and IL-18 and causing electrolyte disruption [15]. This will eventually result in a strong inflammatory response and triggers pyroptosis, a type of inflammatory cell death [15]. 

During OS, the thioredoxin interacting protein (TXNIP) directly binds and activates NLRP3 after dissociating from thioredoxin (TRX) [16]. TXNIP is also referred to as vitamin D3 upregulated protein 1 (VDUP1), a key modulator of the redox system. We have previously reported on the involvement of TXINP in the etiology of tIRI as part of the ASK1/TRX apoptosis pathway [17]. However, its role was not examined in the context of NOX inhibition and its association with the NLRP3 inflammasome. NLRP3 knockout mice subjected to tIRI showed a decreased activation of both inflammatory and apoptotic cascades and preserved spermatogenesis compared to wild-type mice [18]. Other studies have also described a prominent NLRP3 inflammasome expression in IRI of other organs suggesting its prospective therapeutic role for IRI [19]. 

Unraveling the molecular mechanism and crosstalk between testicular OS and inflammation could identify effective therapeutic strategies. In tIRI, targeting a key source of intracellular ROS generation is a plausible option. Therefore, this study aims to inhibit NOX activity in an in vivo rat model of tIRI using its specific inhibitor, apocynin. We hypothesize that NOX inhibition could diminish ROS accumulation, inhibit downstream NLRP3 inflammasome activation and prevent spermatogenic arrest.

## 2. Materials and Methods

### 2.1. Rat Model of tIRI and Apocynin Treatment

Thirty-six male Sprague Dawley (SD) rats were obtained from Charles River Laboratories (Waltham, MA, USA). The rats were 8-weeks-old, weighing 250–300 g, maintained on a 12-h light/12-h dark cycle, and supplied with food and water ad libitum. The rats were subjected to an intraperitoneal (i.p.) injection of an anesthetic comprised of a mixture of Ketamine (50 mg/kg; Tikam, Hikma Pharmaceuticals, Amman, Jordan) and Xylazine (2 mg/kg; Rompun, Bayer GMP, Germany). The ilioinguinal side was shaved and disinfected with Betadine and 70% ethanol. Three groups (*n* = 12 each) were studied: sham, unilateral tIRI only and tIRI treated with apocynin, a NOX inhibitor. Sham rats underwent a standard ilioinguinal incision at the left side, and the left testis was exposed for 1 h, then placed back in the scrotal sac, followed by incision suturing. The sham animals were allowed to recover for 4 h before sacrifice. In the tIRI only, the testicular ischemic injury was imposed upon the left ipsilateral testis using a straight bulldog clamp with 700 g of pressure. The spermatic cord and artery were clamped for 1 h. Thirty minutes into ischemia, rats were i.p. injected with 250 µL drug vehicle, 10% dimethyl sulfoxide (DMSO). After 1 h of ischemia, the clamp was removed to allow testis reperfusion for 4 h, followed by animal sacrifice. The third group was handled similarly as the tIRI-only group, however, apocynin (50 mg/kg dissolved in 10% DMSO; Selleckchem, Houston, TX, USA) was injected instead of DMSO [9]. In all three groups, the right contralateral testes served as a positive internal control. Harvested testes were stored according to the downstream experiments. 

### 2.2. Histological Analysis

Harvested testes were fixed and embedded in paraffin blocks. Tissue sections (4 µm) were sliced and placed on gelatinized microscopic slides for hematoxylin and eosin (H&E) staining. The stained slides were examined with the Zeiss LSM 700 light microscope (Carl Zeiss Microscopy Ltd. Jena, Germany) and images were taken with varying magnifications (10×, 20×, and 40×). Johnsen scoring was used to evaluate spermatogenesis with images taken at 40× magnification [20]. A score of 1–10 was given to evaluate spermatogenesis in each seminiferous tubule (ST). The scores represent the following cellular profile in STs: 10 for complete spermatogenesis, 9 for disorganized spermatogenesis with many spermatozoa, 8 for only few spermatozoa present, 7 for the lack of spermatozoa and the presence of many spermatids, 6 for only few spermatids present, 5 for the lack of spermatozoa and spermatids and the presence of spermatocytes, 4 for only few spermatocytes present, 3 for only spermatogonia present, 2 for the lack of germ cells, and 1 for the lack of germ cells and Sertoli cells in STs. A total of 20 STs/rats/groups were evaluated.

### 2.3. Western Blot

Total protein was extracted from frozen testicular tissues using the radio-immunoprecipitation assay (RIPA) lysis buffer (Santa Cruz, Dallas, TX, USA) mixed with a cocktail of protease inhibitors and stored at −80 °C for downstream biochemical assays. The protein expression of the NLRP3 pathway components was measured by Western blot. Equal protein concentrations (150–250 µg) were resolved on 12% SDS-PAGE along with a protein ladder. Separated protein bands were blotted onto a PVDF membrane (Bio-Rad, Hercules, CA, USA). Blocked membranes were incubated with the primary antibodies using their respective recommended dilutions (Table 1). Washed PVDF membranes were then incubated with a diluted secondary antibody prior to washing and detection of the targeted protein bands using the ECL reagents (GE Healthcare, Chicago, IL, USA). Band signals were visualized using the Chemidoc imaging system from Bio-Rad (Hercules, CA, USA) and their intensity was calculated by the image lab software (Bio-Rad, Hercules, CA, USA).

### 2.4. Biochemical Assays

#### 2.4.1. Antioxidant Enzymes and Molecules Concentrations

The enzyme activity of superoxide dismutase (SOD) and catalase (CAT) were measured quantitatively using their respective kits from Invitrogen™ (Waltham, MA, USA): superoxide dismutase (SOD) colorimetric activity kit and catalase colorimetric activity kit. Following the manufacturer’s protocol, the activity (U/mL) of antioxidant enzymes was measured in 150 µg of protein samples at 450 nm and 560 nm using standard curves for SOD and CAT, respectively. The total antioxidant capacity (TAC) colorimetric assay kit (BioVision, Milpitas, CA, USA) was used to detect small antioxidant molecules in the presence of a proprietary protein mask. Following the manufacturer’s protocol, the levels of small antioxidants were measured in a 150-µg protein sample using a 96-well plate. The absorbance was plotted at 570 nm as a function of Trolox concentration (nmol).

#### 2.4.2. Inflammation Markers Levels

The activity of myeloperoxidase (MPO), an inflammatory mediator, was measured using its respective colorimetric activity assay kit (BioVision, Milpitas, CA, USA). Following the manufacturer’s protocol, MPO activity (mU/mL) was determined using a 150-µg protein sample, and calculated using a standard curve. The protein concentration (pg/mL) of interleukin 1-beta (IL-1β) and interleukin 18 (IL-18) was measured using their respective rat-specific ELISA kits (Wuhan Fine Biotech Co., Wuhan, Hubei, China) following the manufacturer’s protocol. 

#### 2.4.3. Caspases Activity

The activity of caspases 1 and 8 was measured using their respective colorimetric assay kits purchased from BioVision (Milpitas, CA, USA). Following the manufacturer’s protocol, the caspases activity was measured in 150-µg protein samples at 400 nm. The fold increase in each caspase activity was determined by comparing the results of the tIRI only and tIRI-treated with apocynin with that of the sham.

#### 2.4.4. NADP/NADPH Levels

NOX activity was indirectly measured by calculating the NADP/NADPH ratio in testicular tissue homogenates using the NADP/NADPH quantitation colorimetric kit from BioVision (Milpitas, CA, USA). Following the manufacturer’s protocol, the ratio was measured in 150-µg protein samples aliquoted in a 96-well plate. After enzyme cycling reactions, the NADP and NADPH levels were measured at 450 nm and calculated using a standard curve. The NADP/NADPH ratio was calculated using the following formula: (NADPt—NADPH)/NADPH.

### 2.5. RNA Extraction, Reverse Transcription, and Real-Time PCR

Total cellular RNA was isolated from frozen testicular tissues following the TRIzol method (Invitrogen, Waltham, MA, USA). The high-capacity cDNA reverse transcriptase kit (Applied Biosystems, Foster City, CA, USA) was used for the synthesis of complementary DNA (cDNA) from 2 µg of purified total RNA. Gene expression was measured by real-time PCR for the following genes using their specific Taqman assays: TXNIP (Rn01533891_g1), NLRP3 (Rn04244620_m1), PYCARD (Rn00597229_g1), caspase-1 (Rn00562724_m1), GSDMD (Rn01502557_g1), MMP-9 (Rn00579162_m1), and β-actin (Rn00667869_m1). The QuantStudio™ 5 PCR system software was used to calculate the relative mRNA expression using the 2^−ΔΔCt^ method [21].

### 2.6. Statistical Analysis

Raw data were statistically analyzed using GraphPad Prism Software (GraphPad Software, San Diego, CA, USA). One-way analysis of variance (ANOVA) was used to compare the three experimental groups followed by the Holm-Sidak multiple comparison test. Data are presented as the mean ± standard deviation (SD) and were considered significant if *p*-value < 0.05.

## 3. Results

### 3.1. Antioxidant Effect of NOX Inhibition 

**Figure 1:**
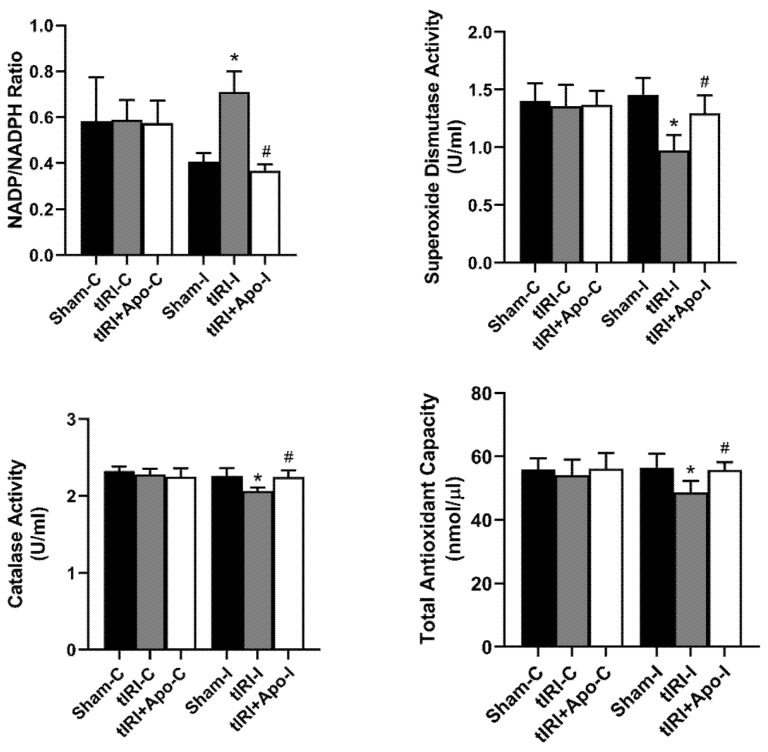
Testicular tissue levels of oxidants and antioxidants. The tissue levels of NADP/NADPH ratio and total antioxidant capacity were measured using colorimetric assays. In addition, the enzyme activities of superoxide dismutase and catalase were determined using their respective colorimetric assay kits. Data are presented as mean ± SD (*n* = 6/group), *p*-value < 0.05. * tIRI compared to sham and ^#^ apocynin-treated compared to tIRI.

During tIRI, an increased ratio of NADP/NADPH by 73.17% (*p*-value = 0.0304) was calculated in ipsilateral testes in comparison to sham (Figure 1). Apocynin treatment normalized the increase in NADP/NADPH ratio suggesting the direct involvement of NOX in generating testicular oxidative stress during tIRI. In testicular tissues of tIRI group, a remarkable decrease of 13.65% (*p*-value = 0.0227), 33.56% (*p*-value = 0.0021), and 8.41% (*p*-value = 0.0042) was calculated for TAC, SOD, and CAT in comparison to the sham group, respectively. Contralateral testes of the three animal groups did not show any significant difference in the NADP/NADPH ratio, TAC, SOD, and CAT (*p*-value > 0.05).

### 3.2. NOX Activates the Expression of the NLRP3 Inflammasome

**Figure 2:**
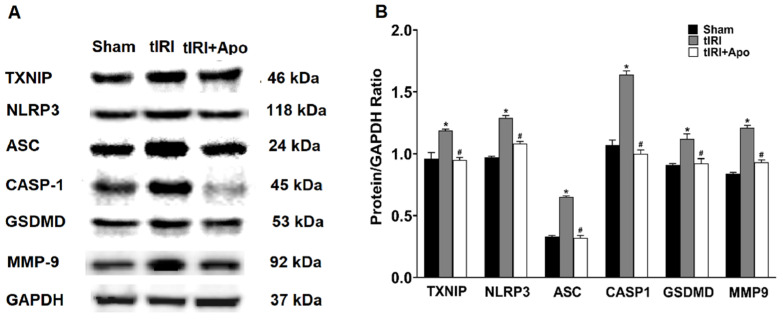
NOX promotes the protein expression of the NLRP3 inflammasome components during tIRI. (**A**) Western blot (WB) detection of NLRP3 proteins’ expression in ipsilateral testes of Sham, tIRI, and tIRI + apocynin (Apo) experimental groups. (**B**) Quantitation of WB band intensity is presented as protein/GAPDH ratio. Data are presented as mean ± SD (*n* = 6/group), *p*-value < 0.05. * tIRI compared to sham and ^#^ apocynin-treated compared to tIRI.

**Figure 3:**
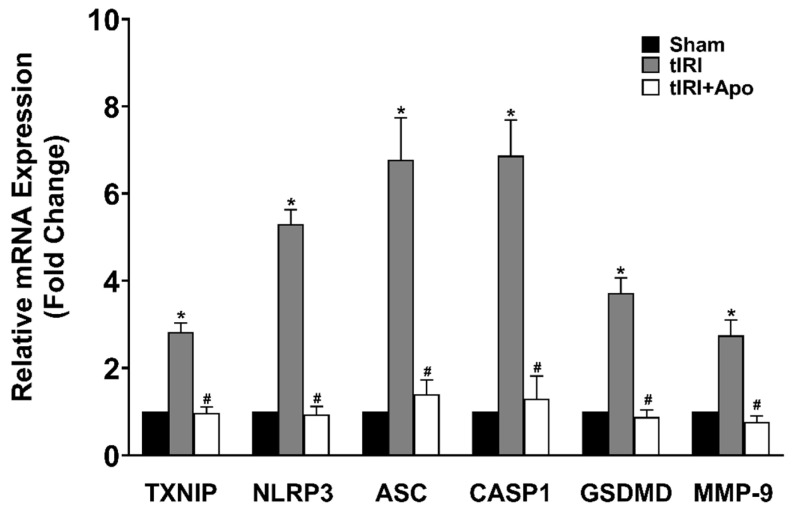
NOX promotes the gene expression of the NLRP3 inflammasome during tIRI in the ipsilateral testes. The relative mRNA expression of the NLRP3 pathway components was measured by the two-step method: reverse transcription (RT) followed by real-time PCR. The fold change in gene expression for tIRI and tIRI + apocynin (Apo) groups was calculated in relation to sham group using the 2^−ΔΔCt^ method. Data are presented as mean ± SD (*n* = 6/group), *p*-value < 0.05. * tIRI compared to sham and ^#^ apocynin-treated compared to tIRI.

Activation of the NLRP3 inflammasome was indicated by the increased protein levels of TXNIP by 24% during tIRI in comparison to the sham group (Figure 2). Similarly, there was an overexpression of the NLRP3 components: NLRP3, ASC, and caspase-1 by 33% (*p*-value = 0.0061), 97% (*p*-value < 0.0001), and 53% (*p*-value < 0.0001), respectively, suggesting the formation of the inflammasome complex during tIRI. Consequently, the protein expression of GSDMD and MMP-9, downstream targets of the NLRP3, was also increased by 24% (*p*-value = 0.0009) and 44% (*p*-value = 0.0008), respectively. The above NLRP3 components were also found to be transcriptionally upregulated during tIRI (Figure 3). In comparison to sham, the gene expression of TXNIP, NLRP3, ASC (encoded by the PYCARD gene), CASP1, GSDMD, and MMP-9 were increased by 2.8-, 5.3-, 6.7-, 6.8-, 3.7-, and 2.7-folds, respectively (*p* ≤ 0.05). NOX inhibition by apocynin treatment has normalized the upregulated transcription and pos-transcription expression of the NLRP3 pathway components. There were no changes in the gene expression (mRNA and protein) of the NLRP3 components in the contralateral testes of the three animal groups (*p*-value > 0.05)

### 3.3. NOX-Activated NLRP3 Triggers Germ Cell Inflammatory Response 

**Figure 4:**
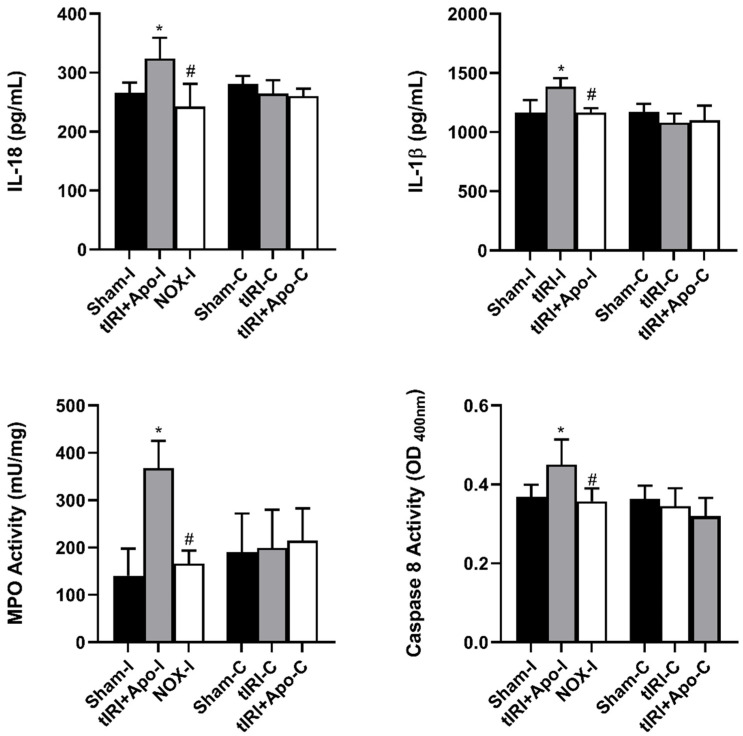
NOX-activated NLRP3 promotes male germ cell inflammatory response. The production of downstream inflammatory targets of NLRP3: caspase 8, IL-1β, and IL-18, was determined by colorimetric assay and ELISA. Caspase 8 activity and cytokine concentrations were calculated following their manufacture’s respective protocols. The data were analyzed using one-way analysis of variance (ANOVA) followed by Holm-Sidak multiple comparisons test and presented as mean values ± SD (*n* = 6/group). * tIRI compared to sham and ^#^ apocynin-treated compared to tIRI. Apo = Apocynin, I = Ipsilateral, C = Contralateral.

Activated NLRP3 triggered the overproduction of the cytokines IL-18 and IL-1β by 22% (*p*-value = 0.0265) and 19% (*p*-value = 0.0123), respectively (Figure 4). In addition, a 114% increase (*p*-value < 0.0001) in the activity of the proinflammatory enzyme MPO was measured during tIRI compared to sham and apocynin-treated rats. Apocynin treatment has prevented the activation of these inflammatory markers. Caspase 8 is known to mediate IL-1β production during inflammation. During tIRI, a 22% increase (*p*-value = 0.007) in caspase 8 activity was recorded compared to sham and apocynin-treated testes. No significant changes were obtained in the contralateral testis for IL-18, IL-1β, MPO, and caspase 8 activity (*p*-value > 0.05).

### 3.4. NOX Induces Spermatogenic Damage 

**Figure 5:**
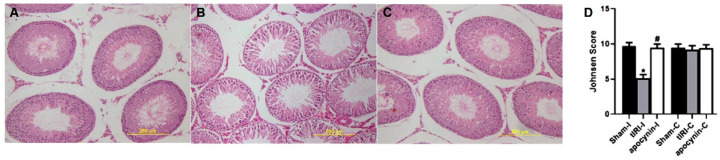
NOX promotes spermatogenic arrest. H&E-stained testicular tissue sections for (**A**) sham, (**B**) tIRI and (**C**) tIRI + apocynin groups were histologically analyzed for spermatogenesis and (**D**) scored using the Johnson score. The tIRI group exhibited impaired spermatogenesis in comparison to sham and apocynin-treated groups that showed normal spermatogenesis. * tIRI compared to sham and ^#^ apocynin-treated compared to tIRI. Apo = Apocynin, I = Ipsilateral, C = Contralateral.

H&E-stained STs were evaluated for spermatogenesis and displayed normal germ cell layers in both sham and apocynin-treated rats (Figure 5). However, tIRI-subjected rats exhibited noticeable ST structural impairment and disrupted germ cell layers indicating the arrest of spermatogenesis. The Johnson scores further confirmed the effect of tIRI on spermatogenesis, which was significantly low in tIRI rats (5.13 ± 0.51) in comparison with sham (9.73 ± 0.46, *p*-value < 0.0001) and apocynin-treated rats (9.47 ± 0.51, *p*-value < 0.0001). A Johnson score of 5 indicates the lack of spermatids and presence of many spermatocytes. Contralateral testes showed no significant changes in the Johnson score (*p*-value > 0.05).

## 4. Discussion

The male reproductive system and the testicular tissue specifically are vulnerable to OS, which makes it a leading factor in the development of male fertility issues [22]. Due to the high NOX expression in the testis, its ROS generation during tIRI or other testicular disease makes it a key culprit in developing testicular OS. A downstream effect of ROS accumulation caused by tIRI is the release of proinflammatory cytokines suggesting the presence of tissue inflammation [22]. This indirect tIRI-induced inflammatory insult may proceed asymptomatically during childhood but could precipitate a fertility issue in adulthood. Such testicular inflammation is termed “sterile” due to the lack of an external pathogen [23]. The results from this study provide evidence for the regulation of the NLRP3 inflammasome system by the tIRI-induced NOX activity.

Although considered a specialized ROS producer, NOX plays a major role in the maintenance of testicular function. Physiologically, NOX is involved in redox regulation during spermatogenesis. NOX-derived ROS is vital for spermatocyte maturation and acrosome formation and capacitation through NOX-dependent apoptosis [24]. Nox1 and Nox3 were proven responsible for increased ROS production leading to reduced self-renewal divisions of mouse spermatogonial stem cells [25,26]. Furthermore, NOX4-generated ROS acted as signaling second messengers that regulate gene expression in male germ cells [27]. The misregulation of NOX activity and excessive ROS generation is equally important. It was related to several male infertility diseases like varicocele, testicular torsion, hypertension, and diabetes [28]. In male infertility, OS could affect the fluidity of the sperm plasma membrane and ROS-induced DNA damage, which may accelerate the process of GCA and decrease sperm count [29,30]. Clinically, there was a NOX5 overexpression in the spermatozoa of asthenozoospermic and teratozoospermic males [6,7]. These conditions were also associated with elevated concentrations of O_2_^−^, H_2_O_2_ and DNA adducts. This clinical impact gave the NOX family a therapeutic appeal. In this study, the histological disappearance of germ cell layers reflects a spermatogenic arrest during tIRI. The disturbed ST structure was associated with an increased NADP/NADPH ratio, an indirect measure of NOX activity. Apocynin treatment prevented spermatogenic arrest, thus, providing a clear link between NOX-induced ROS and testicular dysfunction during tIRI. Apocynin is a plant-derived medicinal herb that interferes with the assembly of the functional NOX complex [31]. Its inhibitory and therapeutic effect was demonstrated in many cells and organs and commonly utilized as a specific NOX inhibitor in research investigations [32,33,34]. Under physiological conditions, the testis relies on its first-line antioxidant defense enzymes to combat intracellular ROS to protect somatic and germinal cells. The frontline antioxidant enzymes SOD and CAT are the core of the testicular antioxidant defense system [35]. In testicular cells, these enzymes counteract ROS generation and prevent their accumulation. Enzymatically, SOD and CAT can locate primer free radicals, neutralize them, and ultimately decrease the rate of oxidation chain formation reactions. Thus, the antioxidant activity of SOD and CAT is known to prevent numerous ailments [36]. A key molecule in the cellular antioxidation systems is NADPH. For their antioxidant activity, both SOD and CAT rely mainly on the availability of intracellular NADPH. Interestingly, NADPH is tightly bound to CAT and prevents its inactivation by the SOD-generated H_2_O_2_ [37]. A recent discovery showed that SOD-produced H_2_O_2_ stimulates NADPH production to fuel more redox reactions of fatty acids and DNA, thus protecting tissues against OS in favor of cell survival [38]. NADPH is also an essential component of the thioredoxin and glutathione systems [39,40]. In parallel, NOX catalyzes NADPH to generate ROS during OS in numerous pathologies. Depletion of NADPH in NOX reactions generates excessive ROS and diminishes its availability to support the activity of ROS-scavenging enzymes. The dual role of NADPH in cellular antioxidation systems explain the correlated observation of ineffective antioxidant systems and increased NOX-generated ROS [40]. In this study, the tIRI-repressed SOD, CAT, and TAC levels were associated with an increased NADP/NADPH ratio. Apocynin treatment normalized the levels of the antioxidant system enzymes and small antioxidant components. This provides direct evidence that NOX is the leading cause of NADPH depletion, a compromised antioxidant system, and ROS overproduction. Other studies have also confirmed a diminished antioxidant defense system in experimental tIRI, which coincides with the current results [41,42,43].

**Figure 6:**
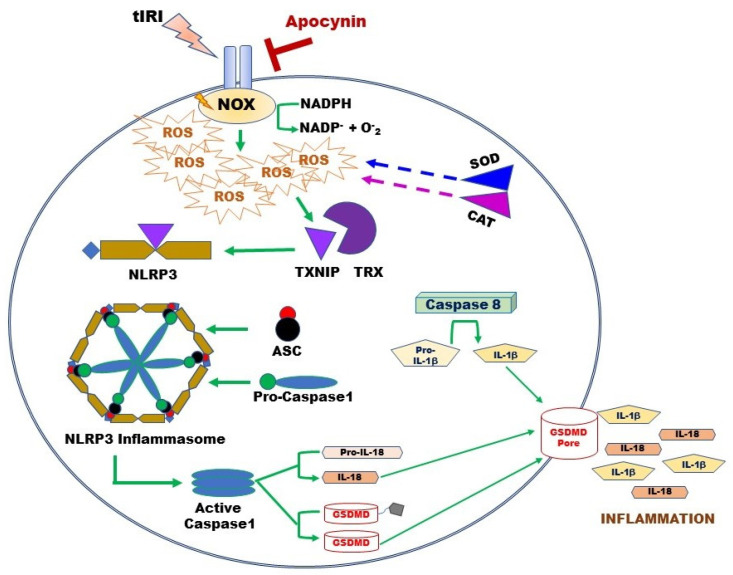
Overview of the activation mechanism of the NLRP3 Inflammasome Pathway during tIRI. Following tIRI, activated NOX sequesters cellular NADPH to produce ROS and thus, weakening the antioxidant enzymes SOD and CAT. ROS accumulation causes TXNIP to dissociate from TRX and binds to NLRP3 protein. Active NLRP3 assembles with ASC and pro-caspase-1 to form the inflammasome. Released caspase-1 will activate IL-18 and GSDMD. Independently, caspase 8 will trigger IL-1β production. GSDMD penetrates the cell membrane, forming non-selective pores to release IL-1β and IL-18 and triggering tissue inflammation. Pore formation results in rapid loss of membrane integrity and lytic cell death.

Besides its negative effect on antioxidant systems during OS, excessive NOX-generated ROS triggers an inflammatory response during tIRI (Figure 6). In non-infectious diseases such as tIRI, the damage-associated molecular patterns (DAMPs) like ROS, OS, and ATP orchestrates the activation of the NLRP3 inflammasome, which causes non-infectious “sterile” inflammation [23]. During tIRI, hyperactive NOX and faulty mitochondria are the two producers of intracellular ROS. Together they activate the canonical NLRP3 inflammasome pathway via priming and activation steps [18,44]. The priming step of the NLRP3 pathway requires the gene expression of NLRP3 and pro-Ilβ, along with the recruitment of ASC [45,46]. The activation step involves the direct binding of TXNIP to the NLRP3 protein [13]. During OS, TXNIP dissociates from TRX and binds NLRP3 leading to inflammasome activation [16]. Upon inflammasome activation, caspase-1 activates proinflammatory cytokines, producing mature IL-1β and IL-18 [47]. Sequentially, IL-1β induces the gene expression of MMP-9, an enzyme involved in extracellular matrix remodeling [48]. Thus, MMP-9 could contribute to tissue fibrosis, a long-term consequence of inflammation, causing potential tissue dysfunction. The active caspase-1 also cleaves GSDMD causing it to form pores in the plasma membrane, which sets off the cell’s osmotic gradient, triggers cell swelling and inflammatory cytokine release, resulting in pyroptosis, an inflammatory form of regulated lytic cell death [49]. Based on the stimulus, caspase-8 can directly regulate IL-1β expression or indirectly through inflammasome regulation [50]. Caspase-8 assembles with ASC and the CARD9–BCL10–MALT1 signalosome, a central proinflammatory signaling complex in innate immune cells. Simultaneously, caspase-8 serves as a positive modulator of the NLRP3-dependent caspase-1 signaling cascades that stimulates both IL-1β production and pyroptotic death [50]. In NOX2 knockout mice, expression of NLRP3 inflammasome components (NLRP3, ASC, caspase-1, and IL-1β) was attenuated in a cerebral cortex injury [51]. Similarly, inhibition of NOX by siRNA or apocynin attenuated the chemically activated NLRP3 via the MAPK and NF-kB pathways [52]. Furthermore, apocynin treatment downregulated NLRP3 expression and improved renal function in a rat model of diabetic nephropathy [53]. Here, we report for the first time that NOX activity regulates the expression of the NLRP3 inflammasome during tIRI. Apocynin treatment abolished this activation.

Several testicular tissue injuries had an association between the NLRP3 inflammasome with cellular and molecular damages. In a rat model of cadmium-induced testicular toxicity, modulation of the NLRP3/caspase-1/IL-1β and the IL-6/STAT3 pathways of inflammation and apoptosis protected male gonads from toxicity [54]. Ning and colleagues also showed that long non-coding RNA could modulate tIRI-induced pyroptosis through the NLRP3/caspase-1 signaling pathway [55]. Using an NLRP3 KO mouse, tIRI caused the overexpression of caspase-1 and cytokines, reduced sperm quality, disintegrated epithelium of STs, and GCA in WT mice compared to KO mice [18]. Furthermore, NLRP3 expression enhanced obesity-associated spermatogenic impairment and male infertility [56]. Lipopolysaccharides (LPS)-activated NLRP3 inflammasome stimulated testicular inflammation by releasing proinflammatory cytokines and impaired testicular function [57]. The same study also demonstrated that an i.p. injection of LPS led to a significant impairment in sperm motility in the epididymis of both wild-type and NLRP3-KO mice. Collectively, NLRP3 inflammasome activation could play a partial role in male subfertility in some pathological and infectious conditions. The findings from these studies are consistent with the results of this study. The current study demonstrates that NOX-activated NLRP3 could participate in tIRI-induced spermatogenic arrest.

## 5. Conclusions

In conclusion, the present study establishes for the first time in the testis that NOX regulates the expression of the NLRP3 components during tIRI. It also suggests the involvement of TXNIP, an OS sensor, in this regulatory mechanism, emphasizing its role in mediating the crosstalk between testicular OS, inflammation, and apoptosis. The parallel occurrence of two types of programmed cell death provides insight into the intriguing, complex, and intertwined cellular network to combat testicular OS in male reproductive disorders. Future studies could utilize NLRP3 inhibitors to explore the role of NLRP3 in tIRI-induced sterile inflammation and spermatogenic arrest. The activation of the NOX/TXNIP/NLRP3 axis during tIRI encourages the pursuit of other possible processes, both inflammatory and non-inflammatory, that generate cytokine and chemokine responses in the testis leading to inflammation-associated male subfertile phenotypes.

## Figures and Tables

**Table 1:** Primary antibodies used for Western blot.

Primary Antibody	Dilution	Manufacturer
NLRP3 (MBS9127062)	1:500	My Bio Source (San Diego, CA, USA)
GAPDH (MBS9131201)	1:10,000	
ASC (sc-514414)	1:500	
Caspase 1 (sc-56036)	1:500	Santa Cruz Biotechnology, Inc. (Dallas, TX, USA)
GSDMDC1 (sc-393581)	1:500	
MMP-9 (sc-393859)	1:500	
VDUP1 (sc-166234)	1:500	

## Data Availability

Data is contained within this article.
